# Supplementary material for: Identification of Key Modules and Candidate Genes for Powdery Mildew Resistance of Wheat-Agropyron cristatum Translocation Line WAT-2020-17-6 by WGCNA
Source: Plants (Basel). 2023 Jan 11;12(2):335. doi: 10.3390/plants12020335 (PMC9864619; doi:10.3390/plants12020335)
Supplement: Supplementary file 1 [file plants-12-00335-s001.zip › Table S1.pdf]

Table S1　Quality statistics of filtered reads

Sample	Raw reads	Clean reads (reads containing joints were removed; Filter to remove low quality data; Remove the N-containing bases)	Clean bases	Error rate	Q20	Q30	GC pct	Total map
C20-0-1	74329944	70901512	10.64	0.02	98.37	95.07	52.57	64786849（91.38%）
C20-0-2	69295888	66645510	10.00	0.02	98.24	94.77	53.23	61227911（91.87%）
C20-0-3	73463226	70133398	10.52	0.02	98.15	94.56	52.84	63376741（90.37%）
K21-0-1	71129920	67837416	10.18	0.02	98.33	95.01	53.81	64008454（94.36%）
K21-0-2	72050682	69105502	10.37	0.02	98.24	94.87	51.83	63473225（91.85%）
K21-0-3	68617620	66581486	9.99	0.02	98.17	94.67	54.73	63275841（95.04%）
C20-6-1	73603672	69372544	10.41	0.02	98.15	94.62	51.93	62625848（90.27%）
C20-6-2	74901032	71097492	10.66	0.02	98.27	94.91	52.24	64232139（90.34%）
C20-6-3	72201846	68808570	10.32	0.02	98.04	94.43	53.24	61655857（89.6%）
K21-6-1	73612678	68599148	10.29	0.02	98.33	95.00	51.04	65109870（94.91%）
K21-6-2	76316924	73296994	10.99	0.02	98.24	94.82	53.12	69454469（94.76%）
K21-6-3	73366606	70271554	10.54	0.02	98.29	94.96	54.41	65488394（93.19%）
C20-12-1	81514510	78904414	11.84	0.02	98.14	94.52	52.75	71670793（90.83%）
C20-12-2	76689442	72639574	10.90	0.02	98.20	94.75	51.82	63344584（87.2%）
C20-12-3	76655910	71779036	10.77	0.03	97.33	93.43	51.13	60943198（84.9%）
K21-12-1	74348258	71084614	10.66	0.02	98.27	94.9	52.65	67261225（94.62%）
K21-12-2	70068074	67146424	10.07	0.02	98.32	95.01	52.66	63255311（94.21%）
K21-12-3	72378562	69272508	10.39	0.02	98.17	94.71	52.81	65499378（94.55%）
C20-24-1	76418740	72224054	10.83	0.02	98.16	94.79	51.83	65076666（90.1%）
C20-24-2	82383450	78901534	11.84	0.02	98.25	94.96	51.79	71327362

								（90.4%）
C20-24-3	78380384	75432404	11.31	0.02	98.15	94.61	52.72	67967240（90.1%）
K20-24-1	71517444	67867966	10.18	0.02	98.25	94.84	52.34	63640713（93.77%）
K20-24-2	76617038	73008600	10.95	0.02	98.27	94.89	53.06	69135945（94.7%）
K20-24-3	72020504	68395702	10.26	0.02	98.2	94.83	51.37	63605715（93.0%）
C20-36-1	69323186	66988008	10.05	0.02	98.06	94.35	52.64	59702275（89.12%）
C20-36-2	73112604	69390404	10.41	0.02	98.26	94.94	52.39	62574354（90.18%）
C20-36-3	73557076	70350926	10.55	0.02	98.26	94.85	52.21	63581884（90.38%）
K21-36-1	78900430	75344944	11.30	0.02	98.18	94.78	52.07	70496878（93.57%）
K21-36-2	74086570	71480784	10.72	0.03	97.89	94.12	52.15	66746889（93.38%）
K21-36-3	77342208	73627756	11.04	0.02	98.27	94.90	51.91	68798628（93.44%）
C20-48-1	75374612	73306024	11.00	0.03	97.90	93.90	51.8	64578124（88.09%）
C20-48-2	74596028	70003078	10.50	0.03	97.14	92.45	51.02	61113137（87.3%）
C20-48-3	71938586	67038458	10.06	0.03	97.28	92.5	51.61	58317953（86.99%）
K21-48-1	74694000	71583886	10.74	0.02	98.19	94.74	51.64	67038491（93.65%）
K21-48-2	72508090	68457424	10.27	0.02	98.17	94.66	51.42	62169062（90.81%）
K21-48-3	71745742	67671280	10.15	0.03	97.2	92.58	51.51	61681869（91.15%）
C20-72-1	71798268	68105932	10.22	0.02	98.06	94.56	51.83	60717952（89.15%）
C20-72-2	69438522	66322256	9.95	0.02	98.18	94.68	51.52	58201923（87.76%）
C20-72-3	71051422	67827946	10.17	0.02	98.24	94.81	51.09	60019186（88.49%）
K21-72-1	76361728	73655514	11.05	0.02	98.26	94.85	51.34	69153612（93.89%）
K21-72-2	87899794	81884098	12.28	0.03	97.71	93.86	50.69	75292296（91.95%）
K21-72-3	74460290	71517908	10.73	0.02	98.31	94.95	52.74	67614580

								（94.54%）
C20-96-1	72532166	69564404	10.43	0.02	98.27	94.86	53.2	61353800（88.2%）
C20-96-2	72121048	69782026	10.47	0.02	98.11	94.50	53.49	62558127（89.65%）
C20-96-3	79864278	76983604	11.55	0.02	98.19	94.72	53.38	69509647（90.29%）
K21-96-1	72347372	69204966	10.38	0.02	98.28	94.96	53.32	64678812（93.46%）
K21-96-2	76162968	71149870	10.67	0.03	97.27	92.54	52.33	65860477（92.57%）
K21-96-3	71923710	68665342	10.30	0.03	97.24	92.54	51.96	63070229（91.85%）
