# Supplementary material for: Identification of Key Modules and Candidate Genes for Powdery Mildew Resistance of Wheat-Agropyron cristatum Translocation Line WAT-2020-17-6 by WGCNA
Source: Plants (Basel). 2023 Jan 11;12(2):335. doi: 10.3390/plants12020335 (PMC9864619; doi:10.3390/plants12020335)
Supplement: Supplementary file 1 [file plants-12-00335-s001.zip › Table S2.pdf]

Table S2 Primer sequences information used for quantitative real-time PCR

Gene ID	Forward primer (5′–3′)	Reverse primer (5′–3′)
TraesCS1A02G225200	GACGAAGGCGATGAAGAA	TCCGACAATGCGGTTATG
TraesCS6A02G281300	GCCTACTACGCCAAATGC	AATCAGCCAGGTTAAGAGC
novel.8020	ATGGCGGTGGATAATAGG	CATAACAGGAGGGAGTGC
TraesCS6D02G008600	CTCCTCGCACATCCATCA	CGTCCACGTACCCGTATTG
novel.10169	TGGTGGATAGTCAGGCTTCT	CGACCCAACATTCAGAGCA
TraesCS1B02G274700	AATGCTGCTGCCGTGCTT	CTTGGTCCTCTTGGAGTTGAT
TraesCS5D02G450500	GCACAGCAAGGCACAACAA	CTTCTGCTCTGGGCTCACC
TraesCS7D02G526100	CCGTGTATGCGAGGATGA	CCAGCAGGGACAAGCAAC
TraesCS5D02G265500	GCCGCTTCGTCTTCTTCA	GGTCTTGCCGTTCTGCTC
TraesCS5B02G426500	GCGTCAGCAGCAGGAACT	GCTGGATGTAGCCCACGAT
TraesCS5A02G332900	GGGAGGAAGATGAGGAGCAGG	TCGGCGATCAGGCGGTAC
novel.7975	GCGGAGGTTCAGAGGATA	TGAGTGGGTGTTGCGTAT
